# Supplementary material for: Differential microRNA expression analyses across two brain regions in Alzheimer’s disease
Source: Transl Psychiatry. 2022 Aug 29;12:352. doi: 10.1038/s41398-022-02108-4 (PMC9424308; doi:10.1038/s41398-022-02108-4)
Supplement: Supplementary file 1 — Supplementary material [file 41398_2022_2108_MOESM1_ESM.docx]

**Supplementary Figure 1**. Box plot displaying the distribution of qPCR-based Ct values for each sample analyzed in this study.


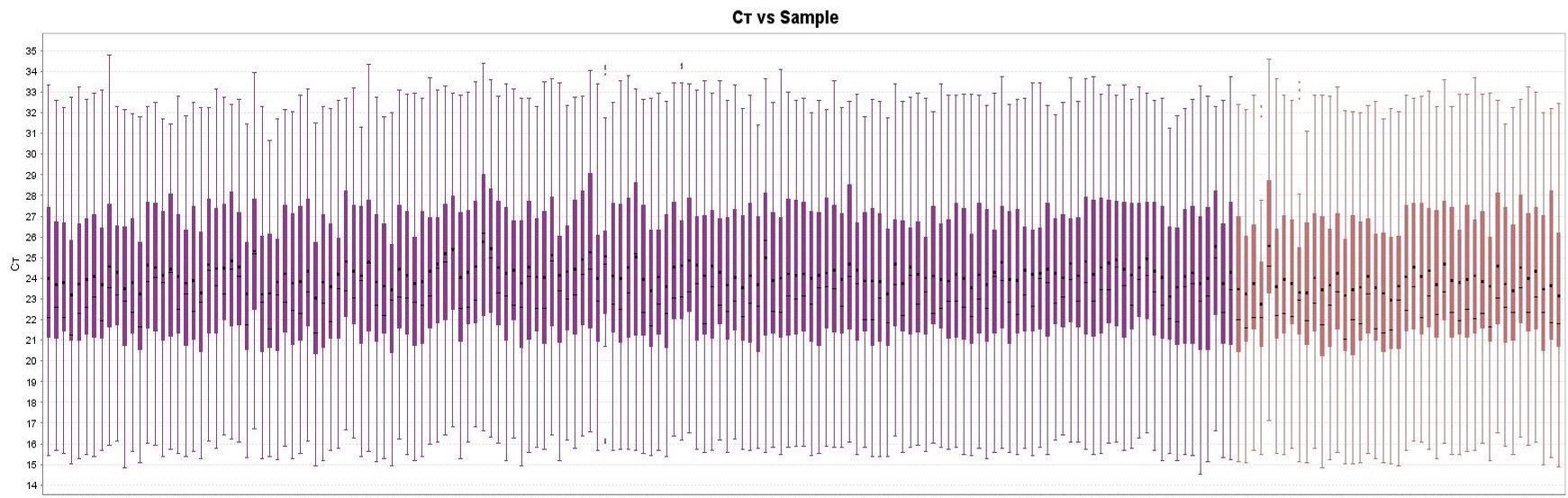


Samples (on x-axis) with RIN<5 are represented in purple and samples with RIN ≥5 in brown color. The solid box shows the range of the middle 50% of the Ct values for each sample. The horizontal black bar shows the median Ct value. The black circle shows the mean Ct value.

**Supplementary Table 1.** Inclusion/exclusion criteria implemented for the literature search on published miRNA differential expression studies in AD.

| **Inclusion criteria** | **Exclusion criteria** |
| --- | --- |
| English language | Not in English language |
| Peer reviewed | Not peer reviewed |
| Comparison of expression levels of miR-125b-5p, miR-129-5p, miR-132-5p, miR-138-5p, miR-195-5p, or miR-501-3p in AD patients vs. control individuals | Off topic / cell models or animal study / no healthy controls |
| Original study | Review / not original article / sample overlap with other studies |
|  | No quantitative results presented (raw data) |

**Supplementary Table 2**. Results of targeted miRNA differential expression analysis in two different brain regions using Braak staging as diagnostic variable.

| miR name (miRBASE) |  | Current study, STG | | | | |  | Current study, EC | | | | |
| --- | --- | --- | --- | --- | --- | --- | --- | --- | --- | --- | --- | --- |
|  |  | Direction | P-value | Effect size (±SE) | CI | N (cases, ctrls) |  | Direction | P-value | Effect size (±SE) | CI | N (cases, ctrls) |
| hsa-miR-125b-5p |  | down | 0.301 | -0.0756 (±0.0729) | -0.218, 0.0672 | 190 (99, 91) |  | up | 0.496 | 0.0465 (±0.0682) | -0.087, 0.180 | 174 (90, 84) |
| hsa-miR-501-3p |  | down | 0.950 | -0.00547 (±0.0874) | -0.177, 0.166 | 174 (89, 85) |  | up | 0.0321 | 0.0804 (±0.0372) | 0.00751, 0.153 | 174 (90, 84) |
| hsa-miR-132-5p |  | down | **2.76E-23** | -0.620 (±0.0538) | -0.726, -0.515 | 190 (99, 91) |  | down | **1.12E-05** | -0.354 (±0.078) | -0.507, -0.201 | 174 (90, 84) |
| hsa-miR-138-5p |  | down | **3.30E-05** | -0.305 (±0.0716) | -0.446, -0.165 | 190 (99, 91) |  | down | **1.10E-04** | -0.0965 (±0.0243) | -0.144, -0.0488 | 174 (90, 84) |
| hsa-miR-195-5p |  | up | 0.169 | 0.105 (±0.0757) | -0.0438, 0.253 | 190 (99, 91) |  | up | **1.30E-04** | 0.191 (±0.0488) | 0.0958, 0.287 | 174 (90, 84) |
| hsa-miR-129-5p |  | down | **9.89E-12** | -0.422 (±0.0579) | -0.536, -0.309 | 190 (99, 91) |  | down | **1.41E-04** | -0.248 (±0.0636) | -0.373, -0.123 | 174 (90, 84) |

STG - superior temporal gyrus; EC- entorhinal cortex; cases – Alzheimer’s disease patients; ctrls - controls; bold font - differential expression reaching significance threshold after multiple testing correction (one-sided test, α = 0.0167; see Methods); SE - standard error; CI - confidence interval.

**Supplementary Table 3**. Results of meta-analyses combining data from Takousis et *al*., new publications identified in our literature search, and the novel data generated in EC brain samples of our project (for meta-analysis results using STG data see Table 2).

| miR name (miRBASE) |  | Direction | P-value | Cases | Ctrls | N total | N studies |
| --- | --- | --- | --- | --- | --- | --- | --- |
| hsa-miR-125b-5p |  | up | **2.80E-08** | 154 | 142 | 296 | 12 |
| hsa-miR-501-3p |  | up | **1.46E-10** | 155 | 153 | 308 | 6 |
| hsa-miR-132-5p |  | down | **9.38E-16** | 144 | 153 | 297 | 7 |
| hsa-miR-138-5p |  | down | **1.68E-10** | 186 | 149 | 335 | 7 |
| hsa-miR-195-5p |  | up | **2.39E-11** | 221 | 196 | 417 | 9 |
| hsa-miR-129-5p |  | down | **5.74E-16** | 644 | 462 | 1106 | 9 |

Cases – Alzheimer’s disease patients; ctrls - controls; bold font - differential expression reaching significance threshold (two-sided test, α = 1.08E-04; see Methods);

**Supplementary Table 4**. Results of targeted mRNA differential expression analysis on top predicted target genes of miRNAs reported in Table 2.

| Ensembl gene ID | Target gene name | miRNA(s) predicted to bind target gene^2^ | FC | P-value | Q-value |
| --- | --- | --- | --- | --- | --- |
| **ENSG00000141526** | ***SLC16A3*** | **hsa-miR-129-5p** | **0.954** | **0.00347** | **0.184** |
| **ENSG00000132002** | ***DNAJB1*** | **hsa-miR-125b-5p** | **0.884** | **0.0323** | **0.404** |
| **ENSG00000111727** | ***HCFC2*** | **hsa-miR-195-5p** | **1.11** | **0.0395** | **0.404** |
| **ENSG00000127083** | ***OMD*** | **hsa-miR-129-5p** | **1.77** | **0.043** | **0.404** |
| **ENSG00000168938** | ***PPIC*** | **hsa-miR-129-5p** | **1.50** | **0.05** | **0.404** |
| ENSG00000158859 | *ADAMTS4^1^* | hsa-miR-129-5p | 0.941 | 0.0502 | 0.404 |
| ENSG00000163288 | *GABRB1* | hsa-miR-138-5p | 0.711 | 0.0534 | 0.404 |
| ENSG00000174469 | *CNTNAP2^1^* | hsa-miR-195-5p | 0.554 | 0.0687 | 0.416 |
| ENSG00000163935 | *SFMBT1* | hsa-miR-138-5p | 0.755 | 0.0706 | 0.416 |
| ENSG00000166006 | *KCNC2* | hsa-miR-125b-5p | 0.541 | 0.0821 | 0.435 |
| ENSG00000181722 | *ZBTB20* | hsa-miR-138-5p, hsa-miR-501-3p | 1.47 | 0.0908 | 0.437 |
| ENSG00000070193 | *FGF10* | hsa-miR-138-5p | 0.97 | 0.131 | 0.478 |
| ENSG00000196862 | *RGPD4* | hsa-miR-195-5p | 0.734 | 0.136 | 0.478 |
| ENSG00000105135 | *ILVBL* | hsa-miR-501-3p | 0.965 | 0.141 | 0.478 |
| ENSG00000073712 | *FERMT2^1^* | hsa-miR-138-5p | 0.985 | 0.143 | 0.478 |
| ENSG00000125520 | *SLC2A4RG* | hsa-miR-129-5p | 1.03 | 0.144 | 0.478 |
| ENSG00000235568 | *NFAM1* | hsa-miR-195-5p | 1.13 | 0.175 | 0.528 |
| ENSG00000177181 | *RIMKLA* | hsa-miR-138-5p | 0.615 | 0.179 | 0.528 |
| ENSG00000137642 | *SORL1^1^* | hsa-miR-125b-5p | 0.899 | 0.206 | 0.558 |
| ENSG00000176407 | *KCMF1* | hsa-miR-501-3p | 0.832 | 0.221 | 0.558 |
| ENSG00000168291 | *PDHB* | hsa-miR-129-5p, hsa-miR-195-5p | 0.757 | 0.221 | 0.558 |
| ENSG00000138443 | *ABI2* | hsa-miR-501-3p | 0.809 | 0.261 | 0.601 |
| ENSG00000143473 | *KCNH1* | hsa-miR-125b-5p, hsa-miR-138-5p | 0.622 | 0.271 | 0.601 |
| ENSG00000169306 | *IL1RAPL1* | hsa-miR-125b-5p | 1.09 | 0.272 | 0.601 |
| ENSG00000124249 | *KCNK15* | hsa-miR-138-5p, hsa-miR-195-5p | 0.798 | 0.286 | 0.605 |
| ENSG00000198087 | *CD2AP^1^* | hsa-miR-195-5p | 1.24 | 0.305 | 0.607 |
| ENSG00000118473 | *SGIP1* | hsa-miR-195-5p | 0.679 | 0.314 | 0.607 |
| ENSG00000068489 | *PRR11* | hsa-miR-195-5p | 0.927 | 0.321 | 0.607 |
| ENSG00000144406 | *UNC80* | hsa-miR-501-3p | 0.663 | 0.349 | 0.637 |
| ENSG00000197548 | *ATG7* | hsa-miR-138-5p | 1.05 | 0.394 | 0.671 |
| ENSG00000182985 | *CADM1* | hsa-miR-501-3p | 0.946 | 0.405 | 0.671 |
| ENSG00000169629 | *RGPD8* | hsa-miR-195-5p | 0.923 | 0.418 | 0.671 |
| ENSG00000155052 | *CNTNAP5* | hsa-miR-125b-5p | 0.551 | 0.424 | 0.671 |
| ENSG00000120675 | *DNAJC15* | hsa-miR-138-5p | 1.14 | 0.456 | 0.671 |
| ENSG00000183454 | *GRIN2A* | hsa-miR-125b-5p | 0.678 | 0.472 | 0.671 |
| ENSG00000120899 | *PTK2B^1^* | hsa-miR-125b-5p | 0.597 | 0.49 | 0.671 |
| ENSG00000066557 | *LRRC40* | hsa-miR-129-5p | 0.777 | 0.498 | 0.671 |
| ENSG00000108406 | *DHX40* | hsa-miR-501-3p | 1.14 | 0.504 | 0.671 |
| ENSG00000185630 | *PBX1* | hsa-miR-129-5p | 0.928 | 0.512 | 0.671 |
| ENSG00000163002 | *NUP35* | hsa-miR-129-5p | 1.01 | 0.522 | 0.671 |
| ENSG00000147439 | *BIN3* | hsa-miR-129-5p | 1.01 | 0.533 | 0.671 |
| ENSG00000132600 | *PRMT7* | hsa-miR-138-5p | 0.764 | 0.551 | 0.671 |
| ENSG00000151612 | *ZNF827* | hsa-miR-125b-5p | 0.984 | 0.556 | 0.671 |
| ENSG00000111602 | *TIMELESS* | hsa-miR-195-5p | 1.32 | 0.557 | 0.671 |
| ENSG00000148082 | *SHC3* | hsa-miR-129-5p | 0.579 | 0.57 | 0.671 |
| ENSG00000140848 | *CPNE2* | hsa-miR-501-3p | 1.30 | 0.617 | 0.711 |
| ENSG00000142192 | *APP^1^* | hsa-miR-138-5p, hsa-miR-195-5p | 0.701 | 0.642 | 0.724 |
| ENSG00000163513 | *TGFBR2* | hsa-miR-501-3p | 1.64 | 0.791 | 0.873 |
| ENSG00000128284 | *APOL3* | hsa-miR-125b-5p | 1.54 | 0.82 | 0.887 |
| ENSG00000183117 | *CSMD1* | hsa-miR-195-5p | 0.701 | 0.863 | 0.915 |
| ENSG00000148331 | *ASB6* | hsa-miR-125b-5p | 0.852 | 0.885 | 0.92 |
| ENSG00000090581 | *GNPTG* | hsa-miR-501-3p | 0.913 | 0.974 | 0.991 |
| ENSG00000026950 | *BTN3A1* | hsa-miR-125b-5p | 1.19 | 0.991 | 0.991 |

1 - Target genes taken from Takousis et al, 2019, all others from TargetScan (see Methods).

2 - Only miRNAs analyzed in this study are listed, other miRNAs not studied here may also bind to the same target gene.

FC: fold change between means of gene aggregated normalized TPMs as calculated by sleuth between AD cases and controls.

P-value: derived from gene level likelihood ratio test (LRT) as calculated by sleuth;

Q-value: P-value adjusted using the Benjamini-Hochberg method to control the false discovery rate (FDR) across all 53 genes assessed

Bold font: miRNAs showing nominally significant evidence of differential expression in AD cases vs. controls.
